# Bis[5-(4-methoxy­benz­yl)furan-3-yl]methanone

**DOI:** 10.1107/S1600536809034333

**Published:** 2009-09-05

**Authors:** Michael Bolte, Lothar Schwarz, A. Stephen K. Hashmi

**Affiliations:** a Institut für Anorganische Chemie, J. W. Goethe-Universität Frankfurt, Max-von-Laue-Strasse 7, 60438 Frankfurt/Main, Germany; b Organisch-Chemisches Institut, Ruprecht-Karls-Universität Heidelberg, Im Neuenheimer Feld 270, 69120 Heidelberg, Germany

## Abstract

The title compound, C_25_H_22_O_5_, was obtained by a dehydrogenative carbonyl­ation reaction. It crystallizes with one half-mol­ecule in the asymmetric unit. The mol­ecules have crystallographic *C*_2_ symmetry and the two atoms of the carbonyl group are located on the rotation axis. The meth­oxy groups are coplanar with the benzene ring to which they are attached [C—C—O—C = 1.0 (6)°]. The two furan rings are inclined at 17.3 (3)° with respect to each other and the dihedral angle between the furan ring and the benzene ring is 75.83 (12)°. The crystal structure is stabilized by C—H⋯O hydrogen bonds.

## Related literature

The palladium-catalysed cyclo­isomerization of allenyl ketones delivers furan derivatives, see: Hashmi (1995 ▶); Hashmi & Schwarz (1997 ▶); Hashmi *et al.* (1999 ▶, 2000 ▶, 2004 ▶); Hashmi, Ruppert, Knöfel & Bats (1997 ▶).
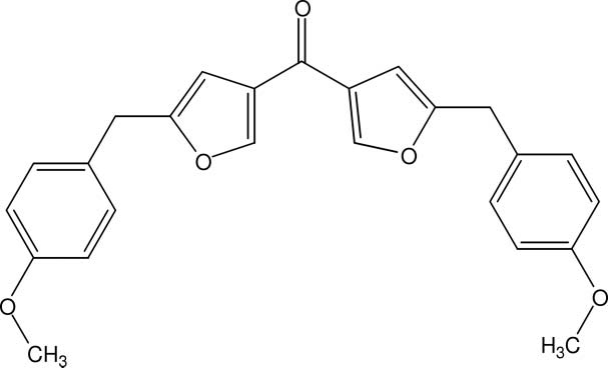


## Experimental

### 

#### Crystal data

C_25_H_22_O_5_
*M* *_r_* = 402.43
Monoclinic, 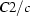

*a* = 42.050 (2) Å
*b* = 5.9183 (2) Å
*c* = 8.3269 (3) Å
β = 99.594 (2)°
*V* = 2043.29 (14) Å^3^
*Z* = 4
Mo *K*α radiation
μ = 0.09 mm^−1^
*T* = 183 K
0.60 × 0.30 × 0.05 mm

#### Data collection

Siemens CCD three-circle diffractometer
Absorption correction: none
8458 measured reflections
1854 independent reflections
1506 reflections with *I* > 2σ(*I*)
*R*_int_ = 0.045

#### Refinement

*R*[*F* ^2^ > 2σ(*F* ^2^)] = 0.081
*wR*(*F* ^2^) = 0.195
*S* = 1.25
1854 reflections
138 parameters
H-atom parameters constrained
Δρ_max_ = 0.31 e Å^−3^
Δρ_min_ = −0.23 e Å^−3^

### 

Data collection: *SMART* (Bruker, 1997 ▶); cell refinement: *SAINT* (Bruker, 1997 ▶); data reduction: *SAINT*; program(s) used to solve structure: *SHELXS97* (Sheldrick, 2008 ▶); program(s) used to refine structure: *SHELXL97* (Sheldrick, 2008 ▶); molecular graphics: *XP* (Sheldrick, 2008 ▶); software used to prepare material for publication: *PLATON* (Spek, 2009 ▶).

## Supplementary Material

Crystal structure: contains datablocks I, global. DOI: 10.1107/S1600536809034333/at2869sup1.cif

Structure factors: contains datablocks I. DOI: 10.1107/S1600536809034333/at2869Isup2.hkl

Additional supplementary materials:  crystallographic information; 3D view; checkCIF report

## Figures and Tables

**Table 1:** Hydrogen-bond geometry (Å, °)

*D*—H⋯*A*	*D*—H	H⋯*A*	*D*⋯*A*	*D*—H⋯*A*
C14—H14⋯O32^i^	0.95	2.23	3.114 (5)	154
C12—H12⋯O15^ii^	0.95	2.87	3.782 (5)	163

Symmetry codes: (i) 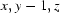; (ii) 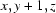.

**Fig. 1.**
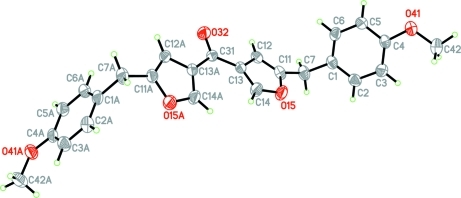
Perspective view of the title compound with the atom numbering; displacement ellipsoids are at the 50% probability level. Symmetry operator for generating equivalent atoms: (A) 1 - x, y, 1/2 - z.

## supplementary crystallographic information

### Comment

The palladium-catalysed cycloisomerization of allenyl ketones delivers furan derivatives (Hashmi, 1995; Hashmi & Schwarz, 1997; Hashmi *et al.*, 1999, 2000, 2004; Hashmi, Ruppert, Knöfel & Bats, 1997). In the context of these investigations, we also conducted the reaction of 1-(4-methoxy-phenyl)penta-3,4-dien-2-one in one atmosphere of carbon monoxide with 0.5 mol% of the PdCl_2_(MeCN)_2_ catalyst in acetonitrile. Besides starting material (7%), the monomeric cyclization product 2-(4-methoxybenzyl)furan (3%) and the cyclization/dimerization product (*E*)-1-[4-methoxybenzyl]-3-{5-[4-(methoxybenzyl]furan-3-yl}but-2-en-1-one (8%) as a new product type the title compound could be isolated (11%). The overall reaction to this new product type is a dehydrogenative carbonylation, mechanistic details are yet unknown.

The title compound crystallizes with half a molecule in the asymmetric unit. The molecules have crystallographic C_2_ symmetry and the two atoms of the carbonyl group are located on the rotation axis. The methoxy groups are coplanar with the phenyl ring to which they are attached [C3—C4—O41—C42 1.0 (6)°]. The two furan rings are inclined by 17.3 (3)° with respect to each other and the dihedral angle between the furan ring and the phenyl ring is 75.83 (12)°. The crystal structure is stabilized by C—H···O hydrogen bonds.

### Experimental

1.30 mmol (245 mg) of 1-(4-methoxy-phenyl)penta-3,4-dien-2-one were dissolved in 7.7 ml MeCN and degassed. The solution was stirred under one atmosphere of CO for two hours, then 6.6 µmol (1.7 mg) Pd(MeCN)_2_Cl_2_ in 0.3 ml MeCN were added. After stirring for 20 h at room temperature the solvent was removed *in vacuo* and the residue was purified by column chromatography on silica gel (eluting with hexanes/ethyl acetate, 5:1). Thus 11% (28.5 mg, 70.8 µmol) of the title compound were obtained. *R*_f_ (H/EE, 5:1) = 0.18. ^1^H NMR (CDCl_3_, 250 MHz): δ = 3.79 (s, 6 H), 3.91 (s, 4 H), 6.42 (d, *J* = 0.9 Hz, 2 H), 6.83- 6.88 (m, 4 H), 7.13–7.20 (m, 4 H), 7.84 (d, *J* = 0.9 Hz, 2 H). ^13^C NMR (CDCl_3_, 62.9 MHz): δ = 33.37 (t, 2 C), 55.14 (q, 2 C), 105.62 (d, 2 C), 113.93 (d, 4 C), 128.16 (s, 2 C), 128.81 (s, 2 C), 129.63 (d, 4 C), 145.69 (d, 2 C), 156.96 (s, 2 C), 158.37 (s, 2 C), 189.94 (s).

### Refinement

H atoms were located in a difference map but finally geometrically positioned and refined using a riding model with fixed individual displacement parameters [U_iso_(H) = 1.2 *U*_eq_(C) or U_iso_(H) = 1.5 *U*_eq_(C_methyl_)] and with C_aromatic_—H= 0.95 Å, C_methyl_—H = 0.98Å and C_methylene_—H = 0.99 Å.

### Crystal data

**Table:**

C_25_H_22_O_5_	*F*(000) = 848
*M**_r_* = 402.43	*D*_x_ = 1.308 Mg m^−^^3^
Monoclinic, *C*2/*c*	Mo *K*α radiation, λ = 0.71073 Å
Hall symbol: -C 2yc	Cell parameters from 5761 reflections
*a* = 42.050 (2) Å	θ = 5.2–24.8°
*b* = 5.9183 (2) Å	µ = 0.09 mm^−^^1^
*c* = 8.3269 (3) Å	*T* = 183 K
β = 99.594 (2)°	Plate, colourless
*V* = 2043.29 (14) Å^3^	0.60 × 0.30 × 0.05 mm
*Z* = 4	

### Data collection

**Table:**

Siemens CCD three-circle diffractometer	1506 reflections with *I* > 2σ(*I*)
Radiation source: fine-focus sealed tube	*R*_int_ = 0.045
graphite	θ_max_ = 26.2°, θ_min_ = 2.0°
ω scans	*h* = −50→51
8458 measured reflections	*k* = −7→7
1854 independent reflections	*l* = −10→9

### Refinement

**Table:**

Refinement on *F*^2^	Secondary atom site location: difference Fourier map
Least-squares matrix: full	Hydrogen site location: inferred from neighbouring sites
*R*[*F*^2^ > 2σ(*F*^2^)] = 0.081	H-atom parameters constrained
*wR*(*F*^2^) = 0.195	*w* = 1/[σ^2^(*F*_o_^2^) + (0.0374*P*)^2^ + 9.4535*P*] where *P* = (*F*_o_^2^ + 2*F*_c_^2^)/3
*S* = 1.25	(Δ/σ)_max_ < 0.001
1854 reflections	Δρ_max_ = 0.31 e Å^−^^3^
138 parameters	Δρ_min_ = −0.23 e Å^−^^3^
0 restraints	Extinction correction: *SHELXL97* (Sheldrick, 2008), Fc^*^=kFc[1+0.001xFc^2^λ^3^/sin(2θ)]^-1/4^
Primary atom site location: structure-invariant direct methods	Extinction coefficient: 0.0039 (8)

### Special details

**Table:**

Geometry. All e.s.d.'s (except the e.s.d. in the dihedral angle between two l.s. planes) are estimated using the full covariance matrix. The cell e.s.d.'s are taken into account individually in the estimation of e.s.d.'s in distances, angles and torsion angles; correlations between e.s.d.'s in cell parameters are only used when they are defined by crystal symmetry. An approximate (isotropic) treatment of cell e.s.d.'s is used for estimating e.s.d.'s involving l.s. planes.
Refinement. Refinement of *F*^2^ against ALL reflections. The weighted *R*-factor *wR* and goodness of fit *S* are based on *F*^2^, conventional *R*-factors *R* are based on *F*, with *F* set to zero for negative *F*^2^. The threshold expression of *F*^2^ > σ(*F*^2^) is used only for calculating *R*-factors(gt) *etc*. and is not relevant to the choice of reflections for refinement. *R*-factors based on *F*^2^ are statistically about twice as large as those based on *F*, and *R*- factors based on ALL data will be even larger.

### Fractional atomic coordinates and isotropic or equivalent isotropic displacement parameters (Å^2^)

**Table:**

	*x*	*y*	*z*	*U*_iso_*/*U*_eq_	
C1	0.37264 (9)	0.2789 (7)	0.4374 (5)	0.0332 (9)	
C2	0.35544 (9)	0.0841 (7)	0.4534 (5)	0.0385 (10)	
H2	0.3643	−0.0267	0.5307	0.046*	
C3	0.32526 (9)	0.0460 (7)	0.3586 (5)	0.0389 (10)	
H3	0.3137	−0.0887	0.3716	0.047*	
C4	0.31251 (9)	0.2064 (7)	0.2458 (5)	0.0328 (9)	
O41	0.28319 (6)	0.1887 (5)	0.1431 (4)	0.0422 (8)	
C42	0.26472 (10)	−0.0101 (9)	0.1577 (6)	0.0534 (13)	
H42A	0.2618	−0.0290	0.2712	0.080*	
H42B	0.2761	−0.1418	0.1237	0.080*	
H42C	0.2436	0.0038	0.0881	0.080*	
C5	0.32947 (9)	0.4037 (7)	0.2275 (5)	0.0363 (10)	
H5	0.3207	0.5142	0.1499	0.044*	
C6	0.35934 (9)	0.4378 (7)	0.3238 (5)	0.0364 (10)	
H6	0.3709	0.5729	0.3114	0.044*	
C7	0.40530 (9)	0.3207 (8)	0.5416 (5)	0.0415 (11)	
H7A	0.4047	0.4680	0.5974	0.050*	
H7B	0.4092	0.2022	0.6265	0.050*	
C11	0.43293 (9)	0.3219 (7)	0.4488 (5)	0.0339 (9)	
C12	0.45192 (8)	0.4804 (7)	0.4054 (4)	0.0308 (9)	
H12	0.4509	0.6373	0.4279	0.037*	
C13	0.47441 (8)	0.3727 (6)	0.3184 (5)	0.0297 (9)	
C31	0.5000	0.4907 (9)	0.2500	0.0297 (12)	
O32	0.5000	0.6987 (7)	0.2500	0.0418 (10)	
C14	0.46659 (10)	0.1524 (7)	0.3128 (6)	0.0418 (11)	
H14	0.4774	0.0387	0.2618	0.050*	
O15	0.44090 (7)	0.1147 (5)	0.3907 (4)	0.0466 (9)	

### Atomic displacement parameters (Å^2^)

**Table:**

	*U*^11^	*U*^22^	*U*^33^	*U*^12^	*U*^13^	*U*^23^
C1	0.0262 (19)	0.044 (2)	0.032 (2)	0.0024 (17)	0.0115 (16)	−0.0021 (18)
C2	0.034 (2)	0.045 (3)	0.037 (2)	0.0054 (18)	0.0087 (18)	0.0062 (19)
C3	0.035 (2)	0.039 (2)	0.045 (2)	−0.0023 (18)	0.0119 (19)	0.005 (2)
C4	0.0239 (18)	0.041 (2)	0.035 (2)	0.0018 (16)	0.0100 (16)	−0.0027 (18)
O41	0.0297 (14)	0.0454 (18)	0.0502 (18)	−0.0019 (13)	0.0032 (13)	0.0012 (14)
C42	0.040 (2)	0.054 (3)	0.063 (3)	−0.014 (2)	−0.002 (2)	−0.003 (3)
C5	0.032 (2)	0.036 (2)	0.041 (2)	0.0020 (17)	0.0063 (18)	0.0038 (18)
C6	0.031 (2)	0.037 (2)	0.044 (2)	−0.0043 (17)	0.0144 (18)	−0.0009 (19)
C7	0.030 (2)	0.060 (3)	0.036 (2)	0.000 (2)	0.0095 (17)	−0.001 (2)
C11	0.0252 (19)	0.045 (2)	0.031 (2)	0.0035 (17)	0.0023 (16)	−0.0062 (18)
C12	0.0280 (19)	0.035 (2)	0.028 (2)	0.0045 (16)	0.0011 (16)	−0.0044 (17)
C13	0.0245 (18)	0.030 (2)	0.035 (2)	0.0011 (16)	0.0040 (15)	0.0000 (17)
C31	0.028 (3)	0.026 (3)	0.035 (3)	0.000	0.004 (2)	0.000
O32	0.040 (2)	0.031 (2)	0.055 (3)	0.000	0.012 (2)	0.000
C14	0.036 (2)	0.032 (2)	0.063 (3)	0.0020 (18)	0.023 (2)	−0.002 (2)
O15	0.0380 (16)	0.0336 (17)	0.074 (2)	−0.0051 (13)	0.0252 (15)	−0.0007 (15)

### Geometric parameters (Å, °)

**Table:**

C1—C2	1.380 (6)	C6—H6	0.9500
C1—C6	1.384 (6)	C7—C11	1.498 (5)
C1—C7	1.518 (5)	C7—H7A	0.9900
C2—C3	1.397 (6)	C7—H7B	0.9900
C2—H2	0.9500	C11—C12	1.321 (6)
C3—C4	1.380 (6)	C11—O15	1.380 (5)
C3—H3	0.9500	C12—C13	1.433 (5)
C4—O41	1.383 (5)	C12—H12	0.9500
C4—C5	1.390 (6)	C13—C14	1.344 (6)
O41—C42	1.426 (5)	C13—C31	1.474 (4)
C42—H42A	0.9800	C31—O32	1.231 (7)
C42—H42B	0.9800	C31—C13^i^	1.474 (4)
C42—H42C	0.9800	C14—O15	1.368 (5)
C5—C6	1.388 (5)	C14—H14	0.9500
C5—H5	0.9500		
			
C2—C1—C6	118.4 (4)	C5—C6—H6	119.3
C2—C1—C7	121.3 (4)	C11—C7—C1	114.3 (3)
C6—C1—C7	120.3 (4)	C11—C7—H7A	108.7
C1—C2—C3	121.5 (4)	C1—C7—H7A	108.7
C1—C2—H2	119.3	C11—C7—H7B	108.7
C3—C2—H2	119.3	C1—C7—H7B	108.7
C4—C3—C2	119.1 (4)	H7A—C7—H7B	107.6
C4—C3—H3	120.4	C12—C11—O15	110.0 (3)
C2—C3—H3	120.4	C12—C11—C7	134.5 (4)
C3—C4—O41	125.1 (4)	O15—C11—C7	115.5 (4)
C3—C4—C5	120.3 (4)	C11—C12—C13	107.6 (4)
O41—C4—C5	114.6 (4)	C11—C12—H12	126.2
C4—O41—C42	116.9 (3)	C13—C12—H12	126.2
O41—C42—H42A	109.5	C14—C13—C12	105.7 (3)
O41—C42—H42B	109.5	C14—C13—C31	129.5 (4)
H42A—C42—H42B	109.5	C12—C13—C31	124.8 (4)
O41—C42—H42C	109.5	O32—C31—C13^i^	118.3 (2)
H42A—C42—H42C	109.5	O32—C31—C13	118.3 (2)
H42B—C42—H42C	109.5	C13^i^—C31—C13	123.4 (5)
C6—C5—C4	119.4 (4)	C13—C14—O15	110.5 (3)
C6—C5—H5	120.3	C13—C14—H14	124.8
C4—C5—H5	120.3	O15—C14—H14	124.8
C1—C6—C5	121.3 (4)	C14—O15—C11	106.2 (3)
C1—C6—H6	119.3		
			
C6—C1—C2—C3	−0.2 (6)	C1—C7—C11—O15	−70.6 (5)
C7—C1—C2—C3	179.5 (4)	O15—C11—C12—C13	−1.9 (4)
C1—C2—C3—C4	0.4 (6)	C7—C11—C12—C13	179.3 (4)
C2—C3—C4—O41	179.1 (4)	C11—C12—C13—C14	1.3 (5)
C2—C3—C4—C5	−0.3 (6)	C11—C12—C13—C31	−179.5 (3)
C3—C4—O41—C42	1.0 (6)	C14—C13—C31—O32	170.0 (4)
C5—C4—O41—C42	−179.6 (4)	C12—C13—C31—O32	−9.0 (4)
C3—C4—C5—C6	0.0 (6)	C14—C13—C31—C13^i^	−10.0 (4)
O41—C4—C5—C6	−179.4 (3)	C12—C13—C31—C13^i^	171.0 (4)
C2—C1—C6—C5	−0.1 (6)	C12—C13—C14—O15	−0.2 (5)
C7—C1—C6—C5	−179.8 (4)	C31—C13—C14—O15	−179.3 (3)
C4—C5—C6—C1	0.2 (6)	C13—C14—O15—C11	−0.9 (5)
C2—C1—C7—C11	113.0 (4)	C12—C11—O15—C14	1.8 (5)
C6—C1—C7—C11	−67.3 (5)	C7—C11—O15—C14	−179.2 (3)
C1—C7—C11—C12	108.1 (5)		

Symmetry codes: (i) −*x*+1, *y*, −*z*+1/2.

### Hydrogen-bond geometry (Å, °)

**Table:**

*D*—H···*A*	*D*—H	H···*A*	*D*···*A*	*D*—H···*A*
C14—H14···O32^ii^	0.95	2.23	3.114 (5)	154
C12—H12···O15^iii^	0.95	2.87	3.782 (5)	163

Symmetry codes: (ii) *x*, *y*−1, *z*; (iii) *x*, *y*+1, *z*.

## References

[bb1] Bruker (1997). *SMART* and *SAINT* Bruker AXS Inc., Madison, Wisconsin, USA.

[bb2] Hashmi, A. S. K. (1995). *Angew. Chem.* **107**, 1749–1751.

[bb3] Hashmi, A. S. K., Choi, J.-H. & Bats, J. W. (1999). *J. Prakt. Chem.* **341**, 342–357.

[bb4] Hashmi, A. S. K., Ruppert, T. L., Knöfel, T. & Bats, J. W. (1997). *J. Org. Chem.* **62**, 7295–7304. 10.1021/jo970837l 11671843

[bb5] Hashmi, A. S. K. & Schwarz, L. (1997). *Chem. Ber. Rec.* **130**, 1449–1456.

[bb6] Hashmi, A. S. K., Schwarz, L. & Bats, J. (2000). *Prakt. Chem.* **342**, 40–51.

[bb7] Hashmi, A. S. K., Schwarz, L. & Bolte, M. (2004). *Eur. J. Org. Chem.* pp. 1923–1935.

[bb8] Sheldrick, G. M. (2008). *Acta Cryst.* A**64**, 112–122. 10.1107/S0108767307043930 18156677

[bb9] Spek, A. L. (2009). *Acta Cryst.* D**65**, 148–155. 10.1107/S090744490804362X PMC2631630 19171970

